# Negative Pressure Wound Therapy versus modified Barker Vacuum Pack as temporary abdominal closure technique for Open Abdomen management: a four-year experience

**DOI:** 10.1186/s12893-017-0281-3

**Published:** 2017-07-21

**Authors:** Giulia Montori, Niccolò Allievi, Federico Coccolini, Leonardo Solaini, Luca Campanati, Marco Ceresoli, Paola Fugazzola, Roberto Manfredi, Stefano Magnone, Matteo Tomasoni, Luca Ansaloni

**Affiliations:** Unit of General Surgery, Papa Giovanni XXIII Hospital, P.zza OMS 1, 24127 Bergamo, Italy

**Keywords:** Temporary abdominal closure, Negative Pressure Wound Therapy, Barker Vacuum Pack, Open Abdomen, Trauma, Emergency surgery

## Abstract

**Background:**

We reviewed our experience with patients presenting with trauma and peritonitis who underwent an open abdomen (OA) procedure, and compared outcomes between Negative Pressure Wound Therapy (NPWT) and a modified Barker Vacuum Pack (mBVP) technique.

**Methods:**

In this descriptive study, we retrospectively analyzed data regarding all patients who underwent OA for intra-abdominal sepsis or abdominal trauma at our Centre from January 2012 to December 2015. Demographic data, co-morbidities, indications to surgery, intra-operative details and Björck classification grade were considered. Outcomes included were: time to closure in days, fascial closure rates, ICU and hospital stay, in-hospital and overall mortality, and entero-atmospheric fistula rate.

**Results:**

A total of 83 cases were considered. Mean closure time was 6 days versus 6.5 days (*p* = 0.71) in NPWT and mBVP groups, respectively; the fascial closure rate was 75.4% versus 93.8% (*p* = 0.10). At multivariate analysis, in-hospital and overall mortality were significantly higher within the mBVP, as compared to NPWT (OR 3.8, 95% CI 1.1 to 13.1, *p* = 0.02 – OR 4.2, 95% CI 1.2 to 14.1, *p* = 0.01). Entero-atmospheric fistula rate was 2.6% in the two groups.

**Conclusions:**

NPWT as a temporary abdominal closure technique, as compared to mBVP, appears to be associated with better outcomes in terms of mortality.

**Electronic supplementary material:**

The online version of this article (doi:10.1186/s12893-017-0281-3) contains supplementary material, which is available to authorized users.

## Background

Open abdomen (OA) has become a commonly-used approach for the management of peritonitis and abdominal trauma in recent years. This technique presents several advantages: it allows the surgeon to treat or prevent intra-abdominal hypertension (IAH) and to manage abdominal compartment syndrome (ACS). In addition, it dramatically decreases the operative time of trauma-related laparotomies, being in line with principles of Damage Control Surgery: bleeding and contamination control, rapidly transfer the patient to Intensive Care Unit for resuscitation. OA also allows to control the source of infection in case of severe intra-abdominal sepsis (IAS) [1, 2] and to plan a ‘second look’ in cases requiring a defined period of monitoring and supporting therapy (e.g. bowel ischemia). However, despite all the above indications, the best technique for temporary abdominal closure (TAC) has not been identified.

Quyn et al. [3] showed that, in absence of IAS, the Wittmann patch along with Vacuum-assisted Pack (VAC) could offer the best outcomes (increased closure rates and decreased mortality and complications). Atema et al. [4], in their analysis on non-traumatized patients, found that Negative Pressure Wound Therapy (NPWT) had the highest fascial closure rates, especially when performed with continuous mesh or suture-mediated fascial traction and dynamic retention sutures; this approach was also related to a low incidence of fistula, which resulted to be equal to 5.7% for patients with fascial closure and NPWT.

In this context, the aim of the present study was to review our experience with the use of OA in patients presenting with intra-abdominal sepsis and abdominal trauma, comparing fascial closure rates and long-term outcomes between patients who received a NPWT versus a modified Barker vacuum pack (mBVP).

## Methods

For the purposes of the current study, we performed a retrospective analysis of all patients who were managed with OA for intra-abdominal sepsis or abdominal trauma from January 2012 to December 2015 at Papa Giovanni XXIII Hospital (Bergamo, Italy). The study was conducted in concordance with the principles of the Declaration of Helsinki. The data were extracted from the institutional Database of Open Abdomen, which is constantly updated at our Centre; the creation of the Database was approved by the Hospital Ethics Committee.

The patients considered in the current study were treated either with a NPWT commercial device (such as ABthera™ Open Abdomen Negative Pressure Therapy device (Kinetic Concepts Inc., San Antonio, TX) [2] and Suprasorb® CNP P1 (Lohmann and Rauscher, Vienna, AU) [5, 6] or with a hand-made vacuum-assisted system; in particular, we used a modified BVP technique, similar to the approach described by Barker et al. [7]. In summary, a perforated polyethylene sheet is placed over intra-peritoneal viscera and beneath the peritoneum of the anterior and lateral abdominal wall. Then, a layer consisting of compressible material, such as sterile surgical gauzes, is applied over the polyethylene sheet. Two silicone drains are then positioned between the gauzes and connected to an aspiration source at −20 cm H2O. The skin surrounding the wound is then dried and covered with the final layer, which consists of a plastic polyester drape.

The indication to NPWT or mBVP was based on the preference of the on-call surgeon at our Centre and on the availability of the devices.

The considered background variables included were demographic characteristics, co-morbidities, surgical indications, intra-operative details, Björck classification grade; the compared outcome were represented by time to closure, fascial closure rates, ICU and hospital stay, in-hospital and overall mortality, entero-atmospheric fistula rate. Intra-hospital mortality was defined in case of death during the same hospitalization as the surgical management; overall mortality inncludes the deaths at any time during follow-up and the intra-hospital mortality.

The main indications for an OA management analyzed in our paper were abdominal trauma and sepsis (intra-abdominal sepsis and intestinal ischemia). In all cases of intra-abdominal contamination, the severity of peritonitis was evaluated according to the Mannheim Peritonitis Index (MPI) at first laparotomy [8]. The severity of trauma was calculated according to the Injury Severity Score (ISS) at arrival in the Emergency Department [9]. OA was classified according to the latest Björck classification system [10].

### Statistical analyses

Continuous variables were expressed as mean ± standard deviation and compared with the Mann-Whitney U test or t-Student test as appropriate. Categorical variables were compared using the Pearson’s Chi square test. Univariate and multivariate models were implemented using binomial logistic regression. A variable was considered significant with *p*-value ≤ 0.05. All analysis was performed using SPSS 23 (IBM Corp. Released 2015. IBM SPSS Statistics for Windows, Version 23.0. Armonk, NY: IBM Corp.).

## Results

Eighty-three patients were included in the study; in particular, 65 patients belonged to the NPWT group, while 18 in the mBVP group. Baseline characteristics of the patients were not significantly different between the groups (Tables 1 and 2). Forty-six patients (55.4%) were male and 37 (44.6%) female; the mean age was 60.9 year ±16.1). The main indications for TAC were sepsis (secondary and tertiary intra-abdominal sepsis and intestinal ischemia) (73 patients, 87.9%) and abdominal trauma (10 patients, 12.1%). More than 90% of patients (*n* = 75) had pre-existing comorbidities and their median ASA score was 4. Mean ISS for traumatized patients was 40.3 (range from 21 to 57). The mean MPI was 26 (range 0–47), and 79.6% of patients (*n* = 66) had a MPI greater than 21.

**Table 1:** Baseline characteristics of the study groups

Variable	General (*N* = 83)	NPWT (*N* = 65)	mBVT (*N* = 18)	*P*-value (NPWT vs mBVT)
Gender, n (%)				
- male - female	46 (55.4) 37 (44.6)	35 (53.8) 30 (46.2)	11 (61.1) 7 (38.9)	0.58
Age (yrs), mean (±SD)	60.9 (±16.1)	63.8 (±14.1)	62.9 (±16.3)	0.82
Diagnosis, n (%)				
- sepsis - trauma	73 (87.9) 10 (12.1)	58 (89.2) 7 (10.8)	15 (83.3) 3 (16.7)	0.49
Comorbidities, n (%)				
- yes - no	75 (90.4) 8 (9.6)	59 (90.8) 6 (9.2)	14 (77.8) 4 (22.2)	0.13
Provenience of pts., n (%)				
- ICU - Ward - ED	20 (24.1) 35 (42.2) 28 (33.7)	15 (23.1) 28 (43.1) 22 (33.8)	5 (27.8) 6 (33.3) 7 (38.9)	0.75
ISS, mean (±SD)^a^	40.3 (±10.7)	41.9 (±12.2)	30.3 (±8.0)	0.18
ASA, mean (±SD)	3.9 (±1)	3.7 (±0.9)	3.7 (±1)	0.90

*NPWT* negative pressure wound therapy, *mBVT* modified Barker Vacuum Technique, *pts.* patients, *SD* standard deviation, *ICU* Intensive Care Unit, *ED* Emergency Department

^a^data from 10 trauma patients

**Table 2:** Intra-operative and post-operative characteristics of the study groups

Variable	General (*N* = 83)	NPWT (*N* = 65)	mBVT (*N* = 18)	*P*-value (NPWT vs mBVT)
Number of changing dressing (n), mean (±SD)	3.3 (±1.9)	3.3 (±1.9)	3.9 (±2.6)	0.22
MPI, mean (±SD)	21.9 (±13.7)	24.4 (±12.2)	28.2 (±9.4)	0.22
- <21, n (%)	17 (20.4)	16 (24.6)	1 (5.6)	
- 21–29, n (%)	33 (39.8)	24 (36.9)	9 (50)	0.19
- >29, n (%)	33 (39.8)	25 (38.5)	8 (44.4)	
Bjork at first laparotomy, n (%)				
- 1a	8 (9.6)	7 (10.8)	1 (5.6)	
- 1b	23 (27.7)	19 (29.2)	4 (22.2)	
- 1c	11 (13.3)	8 (12.3)	3 (16.7)	0.25
- 2a	3 (3.6)	1 (1.5)	2 (11.1)	
- 2b	15 (18.1)	13 (20)	2 (11.1)	
- 2c	21 (25.3)	16 (24.6)	5 (27.8)	
- 3a	1 (1.2)	0 (0)	1 (5.6)	
- 3b	1 (1.2)	1 (1.5)	0 (0)	
Bjork at closure, n (%)^a^				
- 1a	53 (68.8)	40 (65.6)	13 (81.3)	
- 1b	1 (1.3)	1 (1.6)	0 (0)	
- 2a	19 (24.7)	17 (27.9)	2 (12.5)	0. 48
- 2b	2 (2.6)	2 (3.3)	0 (0)	
- 4	2 (2.6)	1 (1.6)	1 (5.6)	
Hospital stay (days), mean (±SD)^a^				
- ICU	22.2 (±18.4)	24.4 (±18.6)	26.9 (±20.7)	0.64
- total	44.3 (±32.5)	47.2 (±31.4)	60.4 (±33.2)	0.14

*NPWT*: negative pressure wound therapy, *mBVT* modified Barker Vacuum Technique, *pts.* patients, *ICU* Intensive Care Unit, *yrs.* years, *SD* standard deviation, *ISS* Injury Severity Score, *MPI* Mannheim Peritonitis Index

^a^data from 77 patients

Intra-operative characteristics and closure data are summarized in Table 2. Dressings were changed a median of 3.3 times (range 1–11).

At first exploration 45.8% of patients (*n* = 38) had a Björck grade Ib and IIb, and 38.6% (*n* = 32) had a grade Ic and IIc (Table 2). At definitive closure 93.5% (*n* = 72) of patients had a clean abdomen, but two developed an entero-atmospheric fistula (EAF) (grade 4), resulting in a fistula rate of 2.6% (Table 2). At closure time, the Björck grade was not significantly different between two groups (*p* = 0.48) (Table 2). Seventy-seven patients (92%) underwent definitive closure of the abdominal wall (Table 3); however, due to the severity of the lesions, it was not possible to close 6 patients who died in ICU (2 traumatic events, 1 hemorrhagic shock, 2 intestinal necrosis/ischemia, 1 septic shock). In 37 (44.6%) patients, a biological or resorbable (Vicryl) mesh was used to facilitate the closure.

**Table 3:** Outcomes

Variable	General (*N* = 83)	NPWT (*N* = 63)	BVT (*N* = 18)	*P*-value (NPWT vs BVT)
Fascial closure rates, n (%)^a^				
- yes - no	61 (79.2) 16 (20.8)	46 (75.4) 15 (24.6)	15 (93.8) 1 (6.3)	0.10
Days to closure (dys), mean (±SD)^b^	5.6 (±4.6)	6 (±4.8)	6.5 (±4.3)	0.71
Intra-hospital mortality, n (%)				
- yes - no	27 (32.5) 56 (67.5)	18 (27.7) 47 (72.3)	9 (50) 9 (50)	0.07
Cause of death				
- Sepsis - Haemorragic shock - Respiratory Failure - Cardiac Failure - MOF - Bowel Ischaemia - Aorto-enteric fistula - Unknown	8 (9.6) 2 (2.4) 2 (2.4) 2 (2.4) 7 (8.4) 1 (1.2) 1 (1.2) 4 (4.8)	4 (6.3) 1 (1.6) 2 (3.2) 1 (1.6) 4 (6.3) 1 (1.6) 1 (1.6) 4 (6.3)	4 (22.2) 1 (5.6) 0 1 (5.6) 3 (16.7) 0 0 0	0.254
Overall mortality, n (%)^c^				
- yes - no	35 (43.8) 45 (56.3)	23 (37.1) 39 (62.9)	12 (66.7) 6 (33.3)	0.02
Cause of death				
- Neoplastic - Recurrent septic episode - Haemorragic shock - Respiratory Failure	4 (4.8) 2 (2.4) 1 (1.2) 1 (1.2)	2 (3.2) 2 (3.2) 1 (1.6) 0	2 (11.1) 0 0 1 (5.6)	0.184

*NPWT* negative pressure wound therapy, *BVT* Barker Vacuum Technique, *pts.* patients, *dys* days, *yrs.* years, *SD* standard deviation, *MOF* Multi-Organ-Failure

^a^data from 77 patients

^b^data from 75 patients

^c^data from 80 patients (3 patients lost at follow up)

No differences were found between groups in terms of mean days to closure (6 days versus 6.5 days; *p* = 0.71) (Table 3). The fascial closure rates were also similar in the two groups (75.4% versus 93.8%; *p* = 0.10) (Table 3).

The mean hospital stay was 44.3 days, with a mean ICU stay of 22.2 days, with no significant differences between the groups (Table 3); data were analyzed excluding 6 patients who died before definitive closure. Overall, in-hospital mortality was 32.5% (Table 3). Data regarding mortality could not be retrieved for 3 patients as they were lost at follow up. A multivariate binary logistic regression model, corrected by age, cause of OA (sepsis or trauma), MPI and ASA score, showed that mBVP was significantly associated with mortality (both in-hospital -OR 3.8, 95% CI 1.1 to 13.1; *p* = .02- and overall mortality -OR 4.2, 95% CI 1.3 to 14.1; *p* = .01) (Table 4).

**Table 4:** Outcomes – Multivariate analysis

Variable (mBVP compared to NPWT)	OR	95% CI	*P*-value
Intra-hospital mortality	3.8	1.1–13.1	0.02
Overall mortality	4.2	1.2–14.1	0.01

*NPWT* negative pressure wound therapy, *BVT* Barker Vacuum Technique

## Discussion

Different techniques for Temporary Abdominal Closure have been proposed; Negative Pressure Techniques (NPT) are considered to be a safer and more effective option than simple skin closure, as they result in drainage of intraperitoneal fluid, rich in toxins and bacteria, allowing approximation of the wound edges at the same time. The two most common NPT approaches are represented by the Barker vacuum pack technique and the vacuum-assisted closure devices [2–4, 7, 11]. NPWT and BVP techniques represent two variants of negative-pressure therapy for the management of Open Abdomen; no exhaustive evidence exists in the Literature to support exclusive use of either device and many Centres utilize the two approaches interchangeably.

Over the last years, a few studies have been conducted to analyze the impact of these techniques on various outcomes, such as fascial closure, fistula rate, mortality rate and costs [4, 11, 12]. The best technique for TAC in case of Open Abdomen has not been univocally identified; the best management is likely to depend on the indication to laparostomy and on the peculiar pathophysiology of every patient.

In the review of our experience, we compared the outcomes of NPWT system versus a hand-made vacuum assisted system (mBVP) and we found no differences in terms of time to closure between the two groups. Our results are in line with those reported by Miller et al. [13] who demonstrated the need for an early fascial closure (within 8 days) in order to significantly reduce complications (derangement of fluids and electrolyte balance, gastrointestinal fistula, adhesions, intra-abdominal infection, respiratory disorders). In our series, the fascial closure rates and the number of dressing changes (mean of 3 changes in both groups) were not statistically different between the two groups.

Atema et al. [4], analyzing only non-traumatized patients, reported that NPWT was associated with the highest fascial closure rate, in particular when associated with continuous mesh or suture-mediated fascial traction (73.1%) and dynamic retention sutures (73.6%). In a prospective study, Batacchi et al. [12] described a significant reduction in time to closure (4.4 versus 6.6 days in Bogotà bag group, *p* = 0.025) and in median ICU and hospital stay (13.3 and 6 days and 28.5 and 21 days respectively) with NPWT techniques. The advantage of reducing ICU length of stay using NPWT was also confirmed by a recent systematic review [14]. In our study we could not find any differences regarding ICU and overall hospital stay; however, these figures seemed to be in favor of NPWT (Table 2).

Overall, the entero-atmospheric fistula (EAF) rate was low in our study (2.6%). This figure is lower than the one reported in the literature (0–15%) [4, 15, 16]. Atema et al. [4] reported lower fistula rates in patients treated with NPWT with fascial closure (5.7%), as compared to NPWT alone (14.6%). In our series patients who developed EAF had severe recurrent or persistent intra-abdominal infections, which represented further life-threatening conditions in view of the prolonged systemic inflammation and the different and resistant microbial flora [3, 17].

The most important differences between NPWT and mBVP groups were seen in the survival-related outcomes. Overall in-hospital mortality in our study was equal to 32.5% and this is in line with previous studies, were this result ranged from 0 to 68% [4]. At multivariate analysis mBVP, as compared to NPWT, showed a significant association with in-hospital and overall mortality (OR 3.8 and 4.2 at multivariate analysis, respectively). Our results are similar to those reported by Kirkpatrick et al. [11] and Cirocchi et al. [14]: in both studies, the Authors demonstrated the superiority of the NPWT systems in terms of reduced 90-day mortality and post-operative mortality [11, 14].

Our study presents some limitations. Firstly, the retrospective non-randomised nature of the statistical analysis may have limited its accuracy. Secondly, mBVP was mainly used at the beginning of our experience with OA and the number of patients who had undergone this technique was limited. Thus, the use of the mBVP group as a comparison group may have lead to a quote of bias. In addition, as suggested by other Authors [18–20], the addition of a retention sutured sequential fascial closure to the mBPV technique might have increased the fascial closure rate.

## Conclusions

Considering the preliminary results presented in the current study, NPWT obtained with commercial devices appears to be associated with better outcomes if compared to the use of the ‘traditional’ TAC methods such as mBVP. Given the retrospective nature of the study and the limited sample size, no definitive conclusions can be drawn; this encourages the need for further study on this topic.

## Additional file

Additional file 1: Data.pdf (PDF 174 kb)

## Abbreviations

ACS: Abdominal compartment syndrome
EAF: Entero-atmospheric fistula
ED: Emergency Department
IAH: Intra-abdominal hypertension
ICU: Intensive Care Unit
ISS: Injury Severity Score
mBVT: Modified Barker Vacuum Technique
MPI: Mannheim Peritoneal Index
NPWT: Negative pressure wound therapy
OA: Open Abdomen
pts.: Patients
SD: Standard deviation
TAC: Temporary abdominal closure
