# Supplementary material for: Negative Pressure Wound Therapy versus modified Barker Vacuum Pack as temporary abdominal closure technique for Open Abdomen management: a four-year experience
Source: BMC Surg. 2017 Jul 21;17:86. doi: 10.1186/s12893-017-0281-3 (PMC5521106; doi:10.1186/s12893-017-0281-3)
Supplement: Additional file 1: — Data.pdf (PDF 174 kb) [file 12893_2017_281_MOESM1_ESM.pdf]

sesso	età	comorbidità	COMORBIDITA
F	56	K RETTO	9
M	55	CIRROTICO	6
M	59	PREGRESSE RESEZ INT MULTIPLE	8
M	78	COLECTOMIA TOT E SPLENECT X	1, 9
M	56	IRC IN DIALISI, ESCARE SACRALI,	2, 5,
M	64	K GASTRICO RECIDIVO SU MONC	8, 9
M	57	PK NECROTICO EMORRAGICA BII	8
M	33	PERICARDITE COSTRITTIVA, SC IN	1, 2
F	66	K OVAIO CON CP, HIPEC ISTEROA	8, 9
M	54	CIRROSI, LE, TOILETTE, SUTURE D	6
F	84	IPT ARTER, BPCO, ARTROSI, OSTE	1, 2,
M	69	PLT DA PRECIPITAZIONE, GIA TRA	0
F	67	IPERT ART, DISTIROID, ASMA, DE	1, 2
F	77	RESEZ ILEALE E PLASTIC ADIRETT	2, 6
M	37	NO	0
F	46	TS, DEPRESSIONE	0
M	75	IPERT ART,SC, DM, K SIGMA CON	1, 2, 3, 8, 9
F	35	K MAMM QUADRANTECTOMIA E	8, 9
M	82	?	0
M	57	EPILESSIA, GST PER ULCERA, AOI	1, 2
M	71	ARTERIOPATICO	2
F	68	IPERTENS ART	2
M	31	APPENDICECTOMIA	0
F	61	K CIECO CON META EPATICHE :E	9
F	72	ESITI DI SD ADERENZIALE. IPERT	2, 9
M	50	CARDIOPATICO, TRAPIANTO CAR	1
M	52	IPERTENS ART	2
M	75	BP AO-BIFEM, INFEZ DI PROTESI,	1, 2
F	45	TETRAPARESI SPASTICA CONNAT	8, 10
M	83	BP AO-BIFEM, CIC, FA, AOMI,	1, 2
F	66	LH STADIO IV	9
F	42	NO	0
M	49	TS, DEPRESSIONE	0
M	73	CIC, BPAC, IPT, TURP, K COLON A	1, 2, 9
M	72	STENOSI AO SEVERA:BPAC IL 18/	1, 2
M	90	BPCO, FIBRILLO FLUTTER, IRC	1, 5
F	78	TIA, ipert art, enfisema, ERNIA JA	1, 2
M	73	HIV	8
M	84	ALZHEIMER	0
M	42	NO	0

F	67	SD rendu osler, CIRROSI CHILD C	6
M	81	SIIFILIDE, AOCP, PREGRESSO POT	1, 2, 3, 6, 8, 9
F	72	SARCOIDOSI, TP CORTISONICA, S	1, 2, 4, 7, 8, 9
M	79	IRC, DM, ISCHEMIA CEREBRALE ,	1, 2, 3, 5
F	75	AR IN TP CORTISON, INSUFF VEN	2, 4, 7
M	62	ARTERIOPATICO, ANEURISMA AO	1, 2
M	46	NO	0
F	18	ATTASSIA TELANGESCTASI, SOSP	7, 8, 9
M	37	NO	0
F	28	TS, DEPRESSIONE	0
M	73	TRAPIANTO CUORE, ERNIA ING S	1,8
M	79	PLASTICA ERNIA ING, IPERT ART,	1, 8, 9
M	79	CARCINOSI PERITON	8, 9
F	79	RECENTE SOSTIT AORTO BIS	2
F	84	NO	0
F	66	AR, IRC IN DIALISI, CORTISONE, IS	7, 5, 2, 10, 4
M	59	EMOPERITONEO DA FRATTURA I	1
F	38	NO	0
F	60	RECENTE RESEZIONE ILEALE PER	11, 2
M	51	CIRROSI, IPERTENSIONE PORTAL	6, 8
F	72	NS	NS
M	81	EMODIALISI CON IRC, IPERTESO,	1, 2, 3, 4, 8
F	61	K OVAIO, 10.3 PERITONECTOMIA	9
M	61	RESEZ COLON TRASV-DISC PER A	2
F	47	K SIGMA CON META EPATICHE ,	8, 9
F	73	STENT A RENALE , ENDOPROTESI	2
M	56	ADK RETTO RECIDIVO, DM, EMI S	3, 8, 9
M	22	DM	3
F	43	GLN DA IGA, IRC, IPERTENSIONE,	2, 5
M	22	NO	0
F	53	K TIROIDE PAPILLIFERO, RTCH CC	9, 8, 4
F	59	K MAMMELLA, ADENOK COLON	9
F	79	FA, OBESITà, PROLASSO UTERINO	1, 3, 5
M	46	CARDIOPATIA,	1

F	66	TX RENE DOPPIO COMPLICATA D	5
M	22	NO	0
M	58	CIRROSI EPATICA E HCC	6, 9
M	75	BILLORTH 2 PER ULCERA, HTA, C	1
F	57	NO	0
F	65	MIELOMA MULTIPLO CON META	4, 8, 9
M	63	PREGRESSO POTUS, K GASTRICO	9
F	52	HIV, HCV, CARDIOPATIA, SD NEF	8, 1, 2, 5
M	68	IPERT ART, COLECISTECTOMIA PA	2
M	58	OBESITA',	2
F	50	K ANO CON COLOSTOMIA E RT, I	9
F	77	K COLON DX INFILTRANTE ILEO E	8, 9
M	57	IMA CON STENT,SD ANSIOSO DE	1
M	69	PREGRESSA RIDUZIONE DI ERNIA	1
M	60		2
M	55	IPERTEN. ART,PKA LUGLIO 2015	1, 2
F	74	IPERTENSIONE ART, PREGRESSO	1
F	68	FA, IPERT ARTERIOSA, IRA, ENCE	1, 2, 3, 6, 9
M	64	IPERT ART, CIRROSI DA FIBROSI C	2,5,6
M	41	ALCOOL	0
M	74	EVAR PER AAA CON STENT AMS	2, 1
F	68	CIRROSI EPATICA SCOMPENSATA	1, 2, 3, 6
M	71	7/12 BYPASS FEMORODISTALE D	1, 2

60,86597938
63
16,10423655

60,83516484
16,50202933

60,98
17,1196152

pz in ICU/reparto/	cause riviste	data 1° intervento
2	1	27/12/11
3	1	21/01/12
2	1	27/01/12
1	1	16/02/12
1	1	01/04/12
1	1	20/04/12
3	1	26/06/12
2	1	11/08/12
2	1	22/08/12
3	2	03/09/12
2	1	12/09/12
1	2	27/09/12
2	1	13/11/12
3	1	28/01/13
3	2	19/02/13
3	1	18/04/13
3	1	07/05/13
2	1	13/05/13
1	1	23/05/13
2	1	18/07/13
2	1	22/07/13
3	1	01/08/13
3	2	06/08/13
2	1	27/08/13
2	1	29/08/13
2	1	13/09/13
1	1	15/11/13
1	1	15/11/13
2	1	26/11/13
3	1	22/12/13
3	1	23/01/14
3	2	05/02/14
3	2	08/03/14
2	1	23/03/14
1	1	08/04/14
3	1	22/04/14
1	1	07/06/14
1	1	21/06/14
1	1	23/06/14
3	2	28/07/14

1	1	04/08/14
1	1	07/08/14
2	1	07/08/14
1	1	28/08/14
2	1	10/09/14
1	1	21/09/14
3	2	27/09/14
3	1	28/09/14
3	2	19/03/14
3	2	02/10/14
2	1	21/10/14
2	1	22/10/14
3	1	05/11/14
1	1	06/11/14
2	1	29/11/14
2	1	01/12/14
3	2	06/12/14
3	2	07/12/14
2	1	08/12/14
3	1	07/01/15
3	2	23/01/15
3	1	11/03/15
3	1	16/03/15
3	1	22/03/15
3	1	23/03/15
2	1	27/03/15
2	1	31/03/15
3	2	11/04/15
3	2	13/04/15
3	2	22/04/15
2	1	25/05/15
2	1	26/05/15
2	1	27/05/15
3	2	05/06/15

1	1	06/06/15
3	2	27/06/15
3	2	30/06/15
1	1	23/07/15
3	2	28/07/15
2	1	31/07/15
2	1	08/08/15
2	1	09/08/15
2	1	25/08/15
3	2	26/08/15
3	1	31/08/15
2	1	03/09/15
3	1	17/09/15
2	1	25/09/15
3	2	05/10/15
2	1	10/10/15
2	1	11/11/15
3	1	17/10/15
3	2	22/11/15
3	2	19/11/15
3	1	05/12/15
1	1	05/12/15
3	1	17/12/15

tipo intervento
LE, LISI ADER, RES ILEAELE, ILEOSTOM LAT, PACKING PELV
LE, TOILETTE, RESEZ E ANAST ILEALE, BOGOTA BAG
LISI ADERENZE,SUTURE VISCERALI, COLOSTOMIA, BOGOTA BAG
LISI ADER, TOILETTE, BOGOTA BAG
EMIDX ALLARGATA AL TRASVERSO, ILEOSTOMIA
TOILETTE, BOGOTA BAG
NECROSECTOMIA, LISI ADERENZE, ABTHERA
RIMOZIONE DI SOSPETTO EMATOMA INFETTO, BOGOTA BAG
RESEZIONE ILEOSOTMIA, RIILEOSTOMIA, BOGOTA BAG
TOILETTE, BOGOTA BAG
RESEZ ILEO CIEC, BOGOTA BAG
TOILETTE, BOGOTA BAG
SUTURA DI PERF DIGIUNALE IN PKA
RESEZ ILEO-CECALE E EMICOLECT DX, ILEOST TERM, RAFFIA DIGIUNO, BOGOTA BAG
LE, RESEZ TENUE, PACKING ADDOME, ABTHERA
GT, BOGOTA BAG
SEZIONE BRIGLIA, RESEZ ILEALE, ABTHERA
RESEZ ILEO-CECALE E OVAIO, ABTHERA
RESEZIONI ILEALI SENZA ANASTOMOSI, BOGOTA BAG
COLECT SX, SPLENECTOMIA, COLOSTOMIA, ABTHERA
RELAPAROTOMIA, ADESIOLISI, RESEZ DIGIUNOILEALE, ILEOST TERM, ABTHERA
TOILETTE, PATCH OMENTALE SU PERF DUOD
RESEZ INTESTIN, PACKING ADDOM, ABTHERA
TOILETTE, RAFFIA ANSA FISTOLIZZATA, ABTHERA
TOILETTE, ABTHERA
TOILETTE, COLEPERITONEO ??, RACCOLTE SACCATE, FEGATO DA SHOCK, PKA, ABTHERA
NECROSECTOMIA, ABTHERA
TOILETTE, ABTHERA
ABTHERA
TOILETTE, RESEZ ILEALE, RIDUZIONE LAPAROCELE, ILEOST TERM, BOGOTA BAG
RESEZIONE ILEALE, ILEOSTOMIA, ABTHERA
LE, SUTURA PERFORAZONI DIGIUNALI, PACKING
PACKING EPATICO
ILEOSTOMIA, ABTHERA
COLECISTECTOMIA
COLECTOMIA SUBTOTALE E ILEOSTOMIA T
RESEZ ILEALE, ANASTOM LL, LAPAROSTOMIA
LE, DRENAGGIO PANCREAS, NECROSECTOMIA, ABTHERA
LE, ABTHERA
PACKING EPATICO, RESEZIONE ILEALE, E COLON, CHIUSURA CUTANEA

RESEZIONE ILEALE, ANASTOMOSI LL, COLECISTECTOMIA, LAPAROSTOMIA
RESEZIONE COLICA SUBTOTALE, ILEOSTOMIA TERMINALE, ANTHERA.
LAVAGGIO E ABTHERA
RESEZIONE ILEALE, EMICOLECTOMIA DX ALLARGATA
SUTURA DEISCENZA ANSA AFFERENTE, SUTURA DUODENALE CON PATCH PROT BIO E PATCH DI AI
SUTURA CON PLADGET DELLA PERF DUOD, DRENAGGIO RACCOLTA RETROP, ABTHERA E VAC
RESEZIONE ILEALE, PACKING RETROPERITONEALE E PELVICO, FISSAZIONE BACINO
APPENDICECTOMIA, ABTHERA
LE PACKING EPATICO, CHIUSURA PARETE
PACKING EPATICO, BOGOTA BAG
LAPARO ESPL, RESEZ ILEO TERMIN, ILEOSTOM, CNP
ADESIOLISI, RESEZ DIGIUNO-ILEALE, PACKING PERITON PER SANGUINAM A NAPPO, LAPAROSTOM
RESEZ ILEALE, ANTHERA
EMICOL SX, LISI ADERENZE, COLOSTOMIA TERM, ABTHERA
LISI SI ADERENZE, LAV, DREN, ABTHERA
RELAPAROTOMIA PER DEISC ANAST ILEO-ILEALE, COLECISTECTOMIA PER COLECISTITE GANGRENO
RELAPAROTOMIA, SUTURA DI PERFORAZIONE GASTRICA E TOILEETTE, ABTHERA
SPLENECTOMIA, PACKING, TPOD, CHIUSURA CUTE
DEISCENZA ANASTOMOTICA, RESEZIONE, MONCONI LIBERI, ABTHERA
TOILETTE, SUTURA DELLA PERFORAZIONE, COLOSTOMIA DETENSIVA SUL TRASVERSO, ILEOSTOMI
PACKING EPATICO, SUTURA COLON, CHIUSURA CUTE
RESEZIOE DIG ILEALE, ABTHERA
SUTURA DEISC ANAST, COLOST LAT, TOILETTE ABTHERA
RELAP, SUTURA DI PERF ILELAE, ABTHERA
LAP ADESIOLISI RAFFIA PERFORAZIONE RETTO COLOSTOMIA LAT ABTHERA
NEFRECTOMIA DX, COLECISTECTOMA, PACKING LOGGIA REN DX ED EPATIO, ABTHERA
RESEZ COLICA, DRENAGGIO ASCESSO PELVICO, PP,
SPLENECTOMIA PACKING CHIUSURA CUTE
RESEZ COLON TRASV, DIGIUNO PROX, SUTURA PERF DIGIUNALE, PACK RETROPERIT SUTURA LOMI
LE, SUTURA DUODENO E POSIZIONAMENTO DI FOLEY, PACKING, DREN TOR
LE, RESEZ 1 MT INTEST, ABTHERA
SUTURA ILEO, LAVAGGIO, ABTHERA
LE, NON ISCHEMIA, ABTHERA
PACKIN ADDOMIN E PACKING EPAT

COLECTOMIA SUBTOT, COLOSTOMIA, ABTHERA
LE, PACKING EPATICO, CHIUSURA CUTE
PACKING EPATICO E ADDOMINALE
DRENAGGIO RACCOLTE RETROPERITON, RAFFIA ILEALE, ABTHERA
PACKING PELVICO E CHIUS CUTE, SPLENECTOMIA, CISTOSTOMIA, PACKING ADDOME CHIUSA CUT
COLECISTECTOMIA LISI ADERENZE, LAPAROSTOMIA
LAVAGGIO E DRENAGGIO, PACKING
RESEZIONE SIGMA, LAVAGGIO, ABBANDONO MONCONI, CNP
LAP, LAVAGGIO, COLECISTECTOMIA, CONTROLLO DUODENO, CNP
LAPARO ESPL SPLENECTOMIA, PACKING EPATICO E DUODEN, DREN TORACICO
LAPAROTOM, RESEZIONE ILEALE, CNP
RELAP, REANASTOMOSI ILEOCECALE
RELAP, LISI ADERENZE, SUTURA DUODEN, TOILETTE RACCOLTA PURULENTA
RELAP, GASTRECT TOTALE, NECROSI ESOF DISTALE, VERSAM PLEURICO E RACCOLTA PARAESOFAG
LAP ESPL PACKING
LAP EXPLO, PERITONITE SIEROPURULENTA, TOILETTE PERITON, ASPORTAZIONE VII-VIII SEGM DI LE
RELAP, TPILETTE, RESEZ ILEALE DUPLICE, SUTURA DI PERF DIGIUNO
RESEZIONE ILEALE 1 MT, CNP
LAP, SINTESI MESO, PACKING, CHIUS CUTE, IAP AL TERMINE 5
LAP, EMOSTASI EPATICA E SPLENICA, SUTURA MESO COLON TRASV, PACKING, CHIUS FASCIA E CU
LAP ESP, ISCHEMIA INTEST DIFFUSA RESEZ COLON SX E SIGMA E ILEO PARZIALE, CNP
LAP ESPL, RESEZIONE ILEO MARCIO, ABTHERA
EMICOLECTOMIA SX, LISI DI ADERENZE, TOILETTE PERITONEALE, COLECISTECTOMIA, CNP, PACKIN

ASA	ISS	open abdomen c	MPI	ORCK ALLA CHIUSU
4		Iia	35	1a
3		Ic	27	1a
3		Iic	28	2a
4		IIa	30	1a
4		Ic	30	1a
3		IIb	32	4
2		IIb	0	1a
4		Ib	25	1a
4		Iic	47	1a
5	21	Iic	34	1a
4		IIb	27	1a
5	35	Ib	26	1a
4		Ic	33	1a morto
4		Iic	39	1a
5	41	Ic	29	2a
1		Ib	22	1b morto
4		IIIa	24	1a
4		Iic	34	1a
2		Ib	22	2a
4		IIb	22	1a
3		Iic	38	2a
2		Iic	30	2b
5	26	Ic	29	1a
4		Iic	36	2a
4		Iic	39	2a
4		IIb	28	1a
2		Ib	22	1a
4		Ib	26	1a
4		Ib	23	1a
4		Iic	27	1a
4		Ib	25	1a
5	26	Iic	34	1a
5	29	Ia	0	1a
4		Iic	27	2a
4		Ib	16	1a
4		Ib	16	1a
4		IIb	43	1a
3		Ib	26	2a
3		Ia	0	1a
5	29	Ic	0	1a

3		Iic	39	1a
4		Ic	34	2a
4		Ib	18	1a
4		Iib	22	1a
4		Iib	33	4
4		Iia	15	1a
5	54	Ia	0	1a
4		Ib	30	1a
5	43	Ia	0	1a
5	35	Ia	0	1a
4		Ib	0	2a
4		IIIb	0	2a
3		Ic	38	1b morto
3		IIb	26	1a
1		IIb	26	1a
4		Ib	33	1a
4	57	IIb	15	1a
5	41	Ia	0	1a
4		Iic	32	2a
3		Ic	23	1a
5	41	Ia	0	1a
5		Ia	0	1a
3		Iib	36	1a
3		Ic	21	1a
4		Iic	25	2a
4		Ia	21	1a
4		Iic	26	2a
5	50	Ia	0	1a
5	50	Iic	17	1a
5	34	Ic	13	1a
4		Iic	34	1a
2		Iic	32	2a
4		Ia	0	1a
5	48	Ia	0	1a morto

4		Ib	23	2a morto
5	38	Ia	0	1a
5	59	Ia	0	1a morto
2		Ic	9	1a
5	48	Ia	0	1a morta
4		Ib	41	1a
3		Ib	23	1a
4		Iic	39	1a
3		Iib	32	2a
5	41	Ia	0	1a morta
3		Ib	32	2a
3		Ib	40	1a
4		Iic	26	1a
3		Ic	22	2a
5	59	Ia	0	1a
2		Iib	28	2a
4		IIc	36	1a
4		Ib	15	1a
5	29	Ia	0	1a
5	41	Ia	0	1A
5		Ib	26	1a
5		Ib	37	1b
4		Iib	34	2a
3,886597938	40,625		21,88659794	
0,956080199	41		26	
	10,92588669		13,69753345	
3,912087912				
0,924380254				
	9,882082552			
0,911603208	33,9			
3,84				
	41,57894737			
	10,10674027			

Temporary Abdon	NUM MEDICAZIONI	tot gg per la chiusura	protesi	tipo protesi (bio/v
2	5	9	2	
2	3	7	2	
2	10	16	1	COLLAMED
2	2	2	2	
2	2	2	2	
2	8	NON CHIUSO		
1	3	5	2	
2	3	6	2	
2	4	9	1	VICRYL
2	2	2	2	
2	4	11	1	VICRYL
2	3	4	2	
2	3	NON CHIUSO		
2	3	4	1	BIODESIGN
1	5	6	2	
2	3	NON CHIUSA		
2	6	13	1	VICRYL
1	3	4	1	COLLAMED
2	2	3	2	
1	4	7	2	
1	4	7	2	
1	10	20	1	SURGISIS
1	4	7	2	
1	4	7	2	
1	4	7	1	SURGISIS
1	3	9	1	VICRYL
1	7	16	1	VICRYL
1	3	4	2	
1	2	2	1	VICRYL
2	4	5	2	
1	3	7	2	
1	4	6	1	PROTEXA
3	2	2	2	
1	4	8	1	PROTEXA
1	3	6	1	SURGISIS
1	3	4	2	
1	5	10	1	PROTEXA
1	6	15	1	PROTEXA
1	2	2	2	
3	2	1	2	

1	2	4	1	VICRYL
1	3	4	2	
1	2	2	1	PROTEXA
1	3	6	1	PROTEXA
1	8	NON CHIUSA	2	
1 + VAC RETROPER	3	5	1	PROTEXA
1	3	3	1	PROTEXA
1	4	7	1	PROTEXA
3	1	2	2	
2	4	4	1	PROTEXA
1	11	30	1	VICRYL
1	5	11	1	PROTEXA
1	1	NON CHIUSO		
1	3	6	1	PROTEXA
1	2	3	2	
1	3	7	1	PROTEXA
1	2	2	2	
3	2	1	2	
1	4	8	2	
1	2	3	1	PROTEXA
3	2	2	2	
1	3	4	2	
1	2	3	2	
1	2	2	1	PROTEXA
1	3	5	1	PROTEXA
1	2	2	2	
1	4	6	1	PROTEXA
3	2	3	2	
3	2	2	1	PROTEXA
3	2	2	2	
1	2	3	2	
1	4	8	1	BIOL
1	4	6	1	VICRYL
1	1	NON CHIUSO		

1	1	NON CHIUSA		
3	3	5	2	
3	2	NON CHIUSO		
1	2	2	2	
3	1	NON CHIUSO		
1	2	3	2	
1	2	2	2	
1	2	2	2	
1	2	2	2	
1	1	NON CHIUSO		
1	4	8	2	
1	2	3	2	
1	2	4	2	
1	5	11	1	PROTEXA
3	2	1	2	
1	2	2	2	
1	3	6	1	PROTEXA
1	2	2	2	
3	2	3	2	
3	6	11	1	PROTEXA
1 + tension	4	6	1	VICRYL
1	2	2	2	
1	3	4	1	VICRYL
	3,268041237			
	3	5,632183908		
	1,895803831	4,585403897		
	3,153846154			
	1,731557275			
	3,8			
	1,958758457			

DIMENS PROTESI	sede	chiusura fascia	2 ..npwt bogota - chiusura fascia
		1	1
		1	1
10*15 E 18*23	3	1	1
		1	1
		1	1
		2	2
		1	1
		1	1
	3	1	1
		1	1
	4	1	1
		1	1
20*30	3	1	1
		1	1
20*30	5	1	1
18*26	5	1	1
		1	1
		1	1
		1	1
	5	1	1
		1	1
		1	1
20*30	3	2	2
	5	1	1
	2	2	2
		1	1
	2	2	2
		1	1
		1	1
	3	1	1
		1	
	3	1	1
20*30	5	1	1
		1	1
20*30	3	2	2
	3	1	1
		1	1
		1	

	5	1	1
		1	1
	1	2	2
25*18	1	1	1
		2	2
	5	2	2
	3	1	1
	5	2	2
		1	
	3	1	1
	2	2	2
20*30	5	2	2
20*30	2	1	1
		1	1
	2	2	2
		1	1
		1	
		1	1
30*20	3	1	1
		1	
		1	1
		1	1
20*30	3	1	1
20*20	3	1	1
		1	1
30*20	5	1	1
		1	
	5	1	
		1	
		1	1
	3	1	1
	1	2	2

		1	
		1	1
		1	1
		1	1
		1	1
		1	1
		1	1
		1	1
		1	1
20*30	2	2	2
		2	
		1	1
20*30	2	2	2
		1	1
		1	
20*30	2	2	
	5	1	1
		2	2
	3	1	1

anastomosi prima	stomia	TIPO
2	1	ILEO
1	2	
1	1	COLO
2	2	
2	1	ILEO
2	2	
2	2	
2	1	ILEO-TRASVERSO
2	2	
1	1	ILEO
2	2	
2	1	ILEO
1	2	
1	1	ILEO
1	2	
2	2	
2	1	COLO
1	1	ILEO
2	2	
1	2	
1	1	ILEO
1	1	BRICKER, DIGIUNO
2	2	
2	1	DIGIUNO
2	2	
2	2	
1	2	
2	1	ILEO
2	2	
2	2	
1	2	
2	2	
1	1	ILEO
1	2	
2	1	DIGIUNO
2	2	
1	2	

1	2	
2	1	ILEO
1	2	
1	2	
1	2	
2	2	
1	2	
1	2	
2	2	
2	2	
1	2	
1	2	
2	1	COLO
2	2	
1	1	COLO
2	2	
2	2	
1	2	
2	1	COLO
2	2	
1	2	
1	1	COLO
2	2	
2	1	COLON TERM
2	2	
2	1	COLON TERM
2	2	
2	2	
1	1	DIGIUNO
2	1	ILEO TERM
1	2	
2	2	

2	2	
1	2	
2	2	
1	2	
2	1	COLON TERM
2	2	
1	1	COLOST
1	2	
2	2	
1	1	DIGIUNO
2	2	
2	2	
1	2	
1	2	
2	2	
2	2	
1	1	COLO
2	2	
2	1	COLO

complicanze	fistola si/no
NO	2
NO	2
DEISC ANASTOM, 29/2 PERITONITE STERCORACEA E EMOPERITON DA SANG	1
NO	1
DEISCENZA FERITA?	1
PKA NECROT EMORR, DEISCENZA ESOF DIGIUN	1
MOF, IRZ E IRESP++	2
NO	2
DEISC FERITA, VAC	2
NO	2
NO	2
NO	2
	1
FISTOLA E-C TTT CONSERVATIVO	2
DEISC FERITA	2
	1
2 FISTOLA ANAST, SD INT CORTO NPT	1
NO	2
NO	1
NO	2
NO	2
DEISC FERITA	2
NO	2
PICCOLA DEISC FERITA	2
SUBOCC INTESTINALI TTT MEDICO	2
NO	2
LAPAROCELE (IRA POST-OP, FISTOLA PK BASSA PORT, INFEZ ADD, DM)	2
DEISC FERITA	1
DEISC FERITA	2
NO	2
NO	2
NO	2
NO	2
INFEZ FERITA	2
NO	1
NO	2
NO	2
NO	2
NO	2
NO	2

NO	2
DEISCENZA FERITA E VAC	2
NO	2
DEISCENZA FERITA PICCOLA, SIERO	2
FISTOLA E-C TTT CONSERVATIVO E VAC	1
NO	1
NO	2
NO	2
NO	2
NO	2
FISTOLA E-CUT, NO SUI PLADGET ALLE ANASTOMOSI	1
DEISC FERITA IMPORTANTE	2
NO	1
NO	1
NO	2
DEISC ANASTOM	1
NO	2
NO	2
FISTOLA ENTERICA BASSA PORTATA RISOLTASI	1
NO	2
NO	2
NO	2
NO	2
NECROSI PARETE CUTANEA , EMATOMA PARTE IN ESITI DI ALLOPLASTICA CC	2
NO	2
NO	2
ECO PARETE PER RACCOLTA SOPRAFASCIALE, DEISC FERITA ZAFFATA	2
NO	2
MEDICAZIONI VAC LOMBARE, INFEZ DA KPC,	2
NO	2
RACCOLTE ADDOMIN TTT CON DREN TC,	2
INFEZ FERITA, FEC, SD INTESTINO CORTO	1
NO	1
	1

	1
NO	2
MORTO IN SALA	1
NO	2
MORTO IN RIA	1
NO	2
FISTOLA BILIARE, EMOPERITONEO REOP IL 25/8, CNP	1
INFEZ FERITA	2
NO	2
	1
SANGUINAM DALL'ANASTOMOSI E PERFORAZ ANAST	1
RITARDO CANALIZZAZIONE, DEISC FERITA	2
RIPRESA FISTOLA DUOD POI CHIUSA SPONTANEAM	2
DEISC ANASTOM, REINTERV PER CERVICOSTOMIA	1
NO	2
NO	2
NO	2
NO	2
NO	1
SD COMPARTIM RISOLTASI, NECROSI FERITA E MINIMO LIQ SIEROSO	2
NO	2
NO	1
SI RIAPERTURE PER SANGUINAMENTO CON REPACKING, RELAPAROSTOMIA,	1

decesso peri op	CAUSA DECESSO	degenza ICU	degenza H (GG)	altro
2		12	71	
1	SEPSI	6	30	
1	SEPSI	28	101	
1	IMA E SHOCK CARI	20	20	
1	SEPSI	10	45	
1	ISCHEMIA ESOFAG	17	20	
2		41	60	LAPAROCELE A 6 M
2		58	117	
2		22	78	A 30 GG RICOVER(
2		7	17	
2		36	98	
2		20	61	
1	SHOCK EMORRAG	16	90	
2	INSUF RESP	71	98	
2		41	98	
1	MOF PER ISCHEMI	4	0	
2		27	53	
2		47	61	
1	mof?	66	69	
2		71	80	
1	COMA	11	7	
2		69	97	
2		10	29	
2		2	65	
2		45	144	
2		40	20	EMODIALISI
2		41	64	
1	SEPSI	2	88	
2		7	46	
1	SEPSI	11	17	
2		23	31	
2		10	18	
2		6	11	
2		19	39	
1	INSUFF RESP	7	48	GASTRECTOMIA T(
1	INFARTO INTESTIN	12	4	
2		73	96	
2		23	42	
2		19	48	
2		37	50	

1		33	43	
2		19	53	
2		37	45	
2		43	74	
2		21	154	
1	FISTOLA AORTICA	20	30	
2		20	39	
2		20	38	
2		10	22	
2		19	72	
2		60	60	
2		16	24	
1	SEPSI	1	0	
1	SEPSI	20	50	
2		4	14	
1	SEPSI DA DEISC AN	10	10	
2		43	57	
2		7	24	
2		21	27	
2		6	20	
2		5	25	
2		21	21	
2		5	43	
2		20	39	
2		10	42	
2		51	69	
2		17	15	
2		10	14	
2		30	58	
2		5	16	
2		6	23	
2		8	38	
1	I RESP	38	58	
1	SHOCK EMORRAG	1	1	

1	MOF	4	4	
2		25	58	
1	ARRESTO CARDIAC	1	1	
2		7	50	
1	CID, ARRESTO CAR	0	0	
2		50	80	
1		29	62	
2		9	28	
2		6	15	
1	SHOCK CARDIOGE	3	3	
2		10	18	
2		21	50	
2		50	57	
1	MOF E SEPSI DA CA	36	66	
2		28	56	
2		4	10	
2		25	98	
2		2	18	
1	nsufficienza epatica	26	26	
2		20	34	
1		39	40	
1	MOF	2	2	
1		17	17	

22,24742268	44,7628866
18,37175761	32,2205205
22,69230769	44,56043956
18,79698103	32,38044979
26,58	56,02
19,72990065	35,10479356

cesso post op/peri	CAUSA DECESSO	data decesso	1 MESE	3 mesi
2				0
0		27/02/12		
0		17/03/12		
0		08/03/12		
0		25/04/12		
0	ima	22/06/11		
1	colecistite acuta	28/08/13		0
2				0
1	PROGRESSIONE K (	21/01/13		DEISC FERITA
2				0
2				LAPAROCELE
2				0
0		20/11/12		
1	INSUF RESP	dic-14		FEC
2				LAPAROCELE
0		23/04/13		
1	PROGRESSIONE K	02/08/13		DECESSO
2				0
0	MOF	05/06/12		
1	EMORRAGIA DIGE:	16/03/14		ricovero per sep
0		11/08/13		
2				LAPAROCELE, FI
2				0
1	PROGRESSIONE K (	nov-14		DEISC FERITA, N
1	PROGRESSIONE K	lug-14		0
2				0
2				12/2/14 PKA
0		23/01/14		
2				LAPAROC
0	SEPSI	02/01/14		
2				RERIC IN MED P
2				0
PERSO				PERSO
2				0
0		26/05/14		
0		09/05/14		
				PERSO
2				LAPROCELE
2				0
2				LAPAROCELE

0		03/10/14		
2				0
2				LAPAROCELE E S
2				0
2				FISTOLA
0		13/10/14		
2				0
1	SHOCK SETTICO	05/09/15		0 RERICOVERO
2				0
2				0
2				FISTOLA EC CON
2				A 2 MESI DEISC
0		06/11/14		
0		08/12/14		
2				0
0		10/12/14		
2				DEISC FERITA
2				0
2				0
2				PERSO
2				PERSO
2	?			NO, DIALISI
2				FA CHTP, LAPAR
2				NON LAPAROCE
2				SEGUITO DAGLI
2				EMODIALISI, PA
2				MEDICAZIONI P
2				0
2				VAC
2				a 1 mese bene,
2				0
2			EVISCERAZ COP	2 MESI DOLORE
0		05/07/15		
0		05/06/15		

0		07/06/15		
2				0
0		01/07/15		
2			PERSO	PERSO
0		28/07/15		
2			NO	0
0	MOF	19/09/15		
2			NO	0
2			NO	0
0		28/08/15		
2			NO	0
2			DEISC FERITA	0
2			NO	0
0	MOF E SEPSI DA C/	02/11/14		
2			RIABILIZAZIONE	RIABILIZAZIONE
2			NO	0
2			NO	0
2			NO	0
0		17/12/15		
2			NO	0
0		13/01/16		
0		07/12/15		
0	SHOCK SETTICO	17/01/16		

**6 MESI**	8 MESI	9 MESI	12 MESI	15 MESI
0	0	0		
laparocele	laparocele	laparocele	laparocele	
0	0	0	0 SC E PERICARI	0
DECESSO				
0	0	0	VIVO, SCOMP ASCITICO	
LAPAROCELE	LAPAROCELE	LAPAROCELE	LAPAROCELE	PERSO
0	0	0	0	0
0	0	0	DECEDUTA	
LAPAROCELE, P	NO	?	?	?
0	0	0	0	0
IVU	DECESSO			
LAPAROCELE, D	LAPAROCELE, D	LAPAROCELE, D	LAPAROCELE, DRENAGGIO ASCI	
0	0	0	0	0
DEISC FERITA, N	DEISC FERITA, N	DEISC FERITA, N	DEISC FERITA, N	DECESSO
0	suboccl int	0	DECESSO	
0	0	0	0	0
0	0	0	laparocele	
LAPAROC	LAPAROC	LAPAROC	LAPAROC	LAPAROC
0	0	0	0	0
0	0	0	0	
PERSO				
RERICOVERO PE	0	0	0	0
PERSO	PERSO	PERSO		
LAPAROCELE	LAPAROCELE	LAPAROCELE	LAPAROCELE	LAPAROCELE
0	0	0	0	
LAPAROCELE	LAPAROCELE	LAPAROCELE	LAPAROCELE	

0				
LAPAROCELE E !	LAPAROCELE E !	LAPAROCELE E !	LAPAROCELE E STIPSI	
0	PERSO	PERSO	PERSO	
FISTOLA	FISTOLA	FISTOLA	FISTOLA EC BENE IL RESTO.	
PERSO	PERSO	PERSO		
0	0	0	DECEDUTA	
0	0	0	0	
0	0	0		
FISTOLA EC CON	FISTOLA EC CON	FISTOLA EC CON	FISTOLA EC CON	FISTOLA EC CON
0	0	0	0	
0	0	0	0	
0	0	0		
0	0	0	0	
0	0	0		
?	?	?		
ROCELE???				
ELE, SIEROMA				
SUBOCCLUSIONI INTESTINALI C(		SUBOCCLUSION	SUBOCCLUSIONI INTESTINALI CON RERICOVERI, CRISI EPILETTICA, META	recidiva, fec
RETE???				
ER DEISC FERITA				
rimossa la digiuno				
0	0	0	0	
EVISCERAZ COP	EVISCERAZ COP	EVISCERAZ COP	EVISCERAZ COPERTA	

0	0	0	0	
PERSO				
PERSO				
0	0			
CON TRACHEO				
0				

18 MESI	21 MESI	24 MESI	27 MESI	30 MESI
		?	?	?
0	0	VIVO		
PERSO	PERSO	PERSO		
0	0	LAPAROCELE, VIVO		
?	?	?		
0	0			
VIVO				
0				
VIVO				
LAPAROC	LAPAROC	LAPAROC		
0	VIVA			
0				

ASCESSO PER ALTRA FISTOLA ENTEROCUTANEA DRENATO, EVISCERAZIONE COP				

			58	

LAPAROCELE SI/NO	time to ventral hernia	FOLLOW UP MAX PER PZ	data 1° intervento
2	53	53	27/12/11
		27/02/12	21/01/12
		17/03/12	27/01/12
		08/03/12	16/02/12
		25/04/12	01/04/12
		22/06/12	20/04/12
1	6	28/08/13	26/06/12
2	44	44	11/08/12
2	44	21/01/13	22/08/12
2	43	43	03/09/12
1	3	43	12/09/12
1	24	43	27/09/12
		20/11/12	13/11/12
2	40	dic-14	28/01/13
1	3	39	19/02/13
		23/04/13	18/04/13
		02/08/13	07/05/13
2	36	36	13/05/13
		05/06/13	23/05/13
2	34	16/03/14	18/07/13
		11/08/13	22/07/13
1	3	33	01/08/13
2	33	33	06/08/13
2	33	nov-14	27/08/13
2	33	lug-14	29/08/13
2	32	32	13/09/13
1	12	30	15/11/13
		23/01/14	15/11/13
1	3	30	26/11/13
		02/01/14	22/12/13
2	28	28	23/01/14
2	27	27	05/02/14
		26	08/03/14
2	26	26	23/03/14
		26/05/14	08/04/14
		09/05/14	22/04/14
		23	07/06/14
1	3	23	21/06/14
2	23	23	23/06/14
1	3	22	28/07/14

		03/10/14	04/08/14
2	21	21	07/08/14
1	3	21	07/08/14
2	21	21	28/08/14
1	3	20	10/09/14
		13/10/14	21/09/14
2	20	20	27/09/14
2	20	05/09/15	28/09/14
2	26	50	19/03/14
2	19	19	02/10/14
1	15	19	21/10/14
2	19	19	22/10/14
		06/11/14	05/11/14
		08/12/14	06/11/14
2	18	18	29/11/14
		10/12/14	01/12/14
2	17	17	06/12/14
2	17	17	07/12/14
2	17	17	08/12/14
		16	07/01/15
		16	23/01/15
2	14	14	11/03/15
1	3	14	16/03/15
2	14	14	22/03/15
2	14	14	23/03/15
2	14	14	27/03/15
2	14	14	31/03/15
2	13	13	11/04/15
2	13	13	13/04/15
2	13	13	22/04/15
2	12	12	25/05/15
1	3	12	26/05/15
		05/07/15	27/05/15
		05/06/15	05/06/15

		07/06/15	06/06/15
2	11	11	27/06/15
		01/07/15	30/06/15
		10	23/07/15
		28/07/15	28/07/15
2	10	10	31/07/15
		19/09/15	08/08/15
2	9	9	09/08/15
2	9	9	25/08/15
		28/08/15	26/08/15
2	9	9	31/08/15
2	8	8	03/09/15
2	8	8	17/09/15
		02/11/15	25/09/15
1	3	7	05/10/15
2	7	7	10/10/15
2	6	6	11/11/15
2	7	7	17/10/15
		17/12/15	22/11/15
2	6	6	19/11/15
		13/01/16	05/12/15
		07/12/15	05/12/15
		17/01/16	17/12/15

17,13114754	media
14	mediana
12,53857436	sd

overall morto si/no	time to status		time to status
2	53		53
1	27/02/12	37	1,2
1	17/03/12	50	1,7
1	08/03/12	21	0,7
1	25/04/12	24	0,8
1	22/06/12	63	2,1
2	46		46
2	44		44
1	44		44
2	43		43
2	43		43
2	43		43
1	20/11/12	7	0,2
1	40		40
2	39		39
1	23/04/13	5	0,2
1	36		36
2	36		36
1	05/06/13	13	0,4
1	34		34
1	11/08/13	20	0,7
2	33		33
2	33		33
1	33		33
1	33		33
2	32		32
2	30		30
1	23/01/14	69	2,3
2	30		30
1	02/01/14	11	0,4
2	28		28
2	27		27
2	26		26
2	26		26
1	26/05/14	48	1,6
1	09/05/14	17	0,6
perso	23		23
2	23		23
2	23		23
2	22		22

1	03/10/14	60	2,0
2	21		21
2	21		21
2	21		21
2	20		20
1	13/10/14	22	0,7
2	20		20
1	20		20
2	26		26
2	19		19
2	19		19
2	19		19
1	06/11/14	1	0,0
1	08/12/14	32	1,1
2	18		18
1	10/12/14	9	0,3
2	17		17
2	17		17
2	17		17
perso	16		16
2	16		16
2	14		14
2	14		14
2	14		14
2	14		14
2	14		14
2	14		14
2	13		13
2	13		13
2	13		13
2	12		12
2	12		12
1	05/07/15	39	1,3
1	05/06/15	0	0,0

1	07/06/15	1	0,0
2	11		11
1	01/07/15	1	0,0
perso	10		10
1	28/07/15	0	0,0
2	10		10
1	19/09/15	42	1,4
2	9		9
2	9		9
1	28/08/15	2	0,1
2	9		9
2	8		8
2	8		8
1	02/11/15	38	1,3
2	7		7
2	7		7
2	6		6
2	7		7
1	17/12/15	25	0,8
2	6		6
1	13/01/16	39	1,3
1	07/12/15	2	0,1
1			
